# Assessing motivation for treatment in eating disorders: psychometric validation of the Italian version of the Autonomous and Controlled Motivation for Treatment Questionnaire (ACMTQ-ITA)

**DOI:** 10.1007/s40519-024-01653-9

**Published:** 2024-04-04

**Authors:** Silvia Tempia Valenta, Matilde Rapezzi, Federica Marcolini, Maurizio Speciani, Gabriele Giordani, Chiara De Panfilis, Diana De Ronchi, Anna Rita Atti

**Affiliations:** 1 Department of Biomedical and Neuromotor Sciences, University of Bologna, Viale Pepoli 5, 40123 Bologna, Italy; 2 Department of Management, University of Bologna, Bologna, Italy; 3 Unit of Neuroscience, Department of Medicine and Surgery, University of Parma, Parma, Italy

**Keywords:** Treatment motivation, Therapeutic motivation, Compliance, Therapy readiness, Treatment adherence, Disordered eating

## Abstract

**Purpose:**

Treatment resistance is a significant challenge in addressing eating disorders (EDs). The Autonomous and Controlled Motivation for Treatment Questionnaire (ACMTQ) has been previously validated in ED populations to assess patients’ motivation for treatment. This study aimed to validate the ACMTQ in the Italian language (ACMTQ-ITA) and evaluate its psychometric properties.

**Methods:**

We recruited a clinical sample of adults aged 18 or older, diagnosed with EDs, proficient in the Italian language, and providing written informed consent. Participants with psychiatric comorbidities such as schizophrenia, bipolar disorder, and substance use disorder were excluded from the study. Validity of the ACMTQ-ITA was assessed using reliability analysis with Cronbach’s α and McDonald’s ω estimates, and Confirmatory Factor Analysis (CFA).

**Results:**

Results from the reliability analysis confirmed the internal consistency of the Autonomous Motivation (AM) factor (α = 0.82, ω = 0.82), the Controlled Motivation (CM) factor (α = 0.76, ω = 0.77), and the ACMTQ-ITA overall score (α = 0.79). The CFA confirmed the two-factor solution (i.e., AM and CM) identified in the original validation of the ACMTQ (Comparative Fit Index = 0.92, Akaike Information Criterion = 3427.26, Bayesian Information Criterion = 3486.82; Root Mean Square Error of Approximation = 0.08, Standardized Root Mean Square Residual = 0.09).

**Conclusion:**

The ACMTQ-ITA emerged as a valid and reliable tool for measuring motivation for treatment in individuals with EDs. Its implementation may facilitate the comprehension of treatment motivation, offering valuable clinical insights and implications for health management practices.

*Level of evidence*: Level V, descriptive studies.

## Introduction

Resistance to treatment poses a significant challenge across various psychiatric disorders [1, 2], encompassing eating disorders (EDs) among them [3–5]. The phenomenon of resistance is quantitatively represented by the occurrence of patient dropout, signifying the unilateral discontinuation of regular treatment [6, 7]. Alarming estimates indicate that treatment dropout rates can escalate to as high as 70% in outpatient settings [8]. This disconcerting trend carries significant clinical implications, such as the likelihood of relapse, prolonged disorder duration, and unfavorable prognosis [9, 10].

In light of these concerns, the accurate evaluation of the patient’s treatment motivation remains a complex issue [11, 12]. Notably, a persistent incongruity often arises between the clinician’s perception of the patient’s motivation for change and the actual experiences reported by the patients themselves [13, 14]. Such discrepancy can lead to the formulation of an inadequate therapeutic approach, carrying significant implications in terms of treatment efficacy [1, 15]. Consequently, there has been a growing interest in employing standardized and reproducible measures to assess motivation, facilitating a more comprehensive understanding of patient responses to treatment interventions.

The Autonomous and Controlled Motivation for Treatment Questionnaire (ACMTQ) stands out as a promising tool initially designed to assess therapy motivation in depression [16]. Originally crafted by Zuroff et al. in 2007, the ACMTQ is a self-report questionnaire grounded in the theoretical constructs of the theory of self-determination [16, 17]. This framework underscores autonomous motivation as a pivotal aspect, wherein individuals perceive their goals as self-selected and self-derived, in contrast to controlled motivation influenced by internal or external influences [18, 19]. Specifically, individuals driven by controlled motivation often feel compelled by factors like guilt or external pressures from others [16, 17, 20].

Subsequently, Mansour and colleagues adapted the ACMTQ in 2012 for patients with bulimic spectrum disorders [21], and later in 2019 Sansfaçon validated its applicability for use in EDs [22]. In both contexts, the ACMTQ demonstrated a notable capacity to predict treatment outcomes, as well as clinical and psychopathological improvements [21, 22]. This attests to its high potential as a valuable instrument for gauging motivation and informing therapeutic interventions in the management of EDs and related conditions.

The aim of our study was to validate the ACMTQ in Italian (ACMTQ-ITA) to offer Italian clinicians a practical tool for assessing motivation in ED patients. We chose the ACMTQ due to its established effectiveness in various psychiatric disorders, including EDs, and its theoretical foundation in the self-determination theory. By validating it in Italian, we aimed to bridge a critical gap in the literature, providing clinicians with a reliable instrument to tailor interventions according to patients’ motivational profiles.

## Materials and methods

### Study participants

Patients were consecutively enrolled on a voluntary basis during their first clinical interview at the Unit of Clinical Psychiatry, Study and Care Unit for ED, Department of Biomedical and Neuromotor Sciences/DIBINEM, University of Bologna, Italy. The inclusion criteria comprised (a) being 18 years or older, (b) having a diagnosis of anorexia nervosa (AN), bulimia nervosa (BN), binge eating disorder (BED), or other specified/unspecified feeding or eating disorders (OSFED/UFED), (c) providing written informed consent, and (d) demonstrating proficiency in the Italian language. Individuals with schizophrenia, bipolar disorder, and substance use disorder were excluded from the study to maintain a more homogeneous sample and enhance the validity of assessing motivation for treatment specifically in individuals with EDs.

### Psychometric measures

The ACMTQ is a self-report questionnaire that investigates motivation for treatment, consisting of 12 items, which determine two subscales of 6 items each [16, 21]. The two subscales assess Autonomous Motivation (AM) and Controlled Motivation (CM) as two independent variables. Sample items are “I feel personally satisfied when I follow my ED treatment” (AM) and “I would be ashamed of myself if I didn’t” (CM). Each question is rated on a 7‐point Likert scale ranging from 1 (strongly disagree) to 7 (strongly agree). Two mean scores are derived, one per subscale (i.e., AM and CM). Higher scores indicate a stronger endorsement of the motivation type.

The Eating Disorder Examination Questionnaire (EDE-Q) is a semi-structured 7-point Likert self-administered test that evaluates body perception and eating habits [23]. It is divided into four subscales (restraint, eating concern, shape concern, and weight concern), reflecting the main features of eating disorders psychopathology [23]. Individuals with a score higher than 2.8 are considered as at high risk of having a clinical ED [23].

The Body Uneasiness Test (BUT) is a self-administered 6-point Likert questionnaire specifically designed to explore several areas in clinical and non-clinical populations: body shape and/or weight dissatisfaction, avoidance, compulsive control behaviors, detachment and estrangement feelings toward one’s own body, specific worries about particular body parts, shapes or functions [24]. A general severity index higher than 1.2 is considered an index of clinically relevant discomfort in one’s own body [25, 26].

### Translation of ACMTQ

The ACMTQ was translated from English into Italian. We used a multi-step forward method to translate the questionnaire: two experimenters (A.R.A. and M.S.) independently translated the questionnaire text and subsequently discussed the drafting to the correct version collegially. The translated questionnaire was then subjected to revision by an English mother-tongue translator for verification and correction and finally approved by a senior researcher (D.D.R.). The Italian version of the ACMTQ questionnaire (ACMTQ-ITA) is available in Appendix for reference.

### Data analysis

Data were analyzed using the Statistical Package for Social Sciences (SPSS) software for macOS (version 26.0, IBM Corp., Armonk, NY, USA, 2019) and R Studio software for macOS (version 1.4.1106). Continuous variables were reported as means ± standard deviation (SD), categorical as frequencies or percentages (*N*; %). The distribution of continuous variables was analyzed using the Shapiro–Wilk normality test.

The validation of the Italian version of ACMTQ (ACMTQ-ITA) consisted of a two-step process. First, we computed Cronbach’s alphas to estimate the internal consistency of the two factors of the ACMTQ scale and the ACMTQ-ITA’s overall score. Additionally, since AM and CM factors comprise six items, we further estimated McDonald’s ω for them. We carried out this analysis using OMEGA Macro for SPSS [27]. Second, we conducted a Confirmatory Factor Analysis (CFA) to assess the Comparative Fit Index (CFI), Akaike Information Criterion (AIC), Bayesian Information Criterion (BIC), Root Mean Square Error of Approximation (RMSEA), and Standardized Root Mean Square Residual (SRMR).

While the study focused on validating the ACMTQ-ITA, the EDE-Q and the BUT questionnaires were also used to comprehensively describe the sample characteristics. Although these measures were not included in the formal analyses, they were employed to provide a detailed profile of the individuals participating in the study, facilitating a better understanding of the severity of ED symptoms and body image concerns within the sample population.

## Results

### Sample description

The key data are thoroughly presented in Table 1. Our initial sample size comprised 94 individuals, but after data cleansing, it was reduced to 80 participants. Ultimately, the study included a total of 80 patients, with 76 identifying as female (95%). The mean age was 28.49-year old (SD = 10.45), with a minimum of 18 and a maximum of 58 years. The mean BMI was 23.09 kg/m^2^ (SD = 7.71), ranging from a minimum of 13.7 kg/m^2^ to a maximum of 47.95 kg/m^2^.

**Table 1:** Sample’s sociodemographic and clinical characteristics

	Minimum	Maximum	Mean ± SD
Age (years)	18	58	28.49 ± 10.45
BMI (kg/m^2^)	13.70	47.95	23.09 ± 7.71
Body uneasiness test (BUT)			
Weight phobia	0.00	5.00	3.21 ± 1.12
Body image concerns	0.00	4.78	2.75 ± 1.20
Avoidance	0.00	4.00	1.59 ± 1.07
Compulsive self-monitoring	0.00	6.00	2.15 ± 1.40
Depersonalization	0.00	4.50	1.88 ± 1.14
Global Severity Index	0.08	4.88	2.44 ± 1.02
Eating Disorder Examination Questionnaire (EDE-Q)			
Restraint	0.00	6.00	3.34 ± 1.69
Eating concern	0.20	5.80	2.66 ± 1.40
Weight concern	0.00	6.00	3.63 ± 1.45
Shape concern	0.30	6.00	4.10 ± 1.40
Total score	0.60	5.70	3.43 ± 1.25
Autonomous and Controlled Motivation for Treatment Questionnaire (ACMTQ-ITA)			
Autonomous motivation	2	7	5.72 ± 1.03
Controlled motivation	1	6.83	3.36 ± 1.41

*SD* standard deviation

Regarding ED diagnoses, among the subjects, 31 (38.8%) were diagnosed with AN, with 14 (17.5%) classified as AN restrictive type and 17 (21.3%) as binge-eating/purging type. Additionally, 26 (32.5%) participants suffered from BN, 16 (20%) from BED, and 7 (8.8%) from OSFED/UFED.

**Fig. 1:**
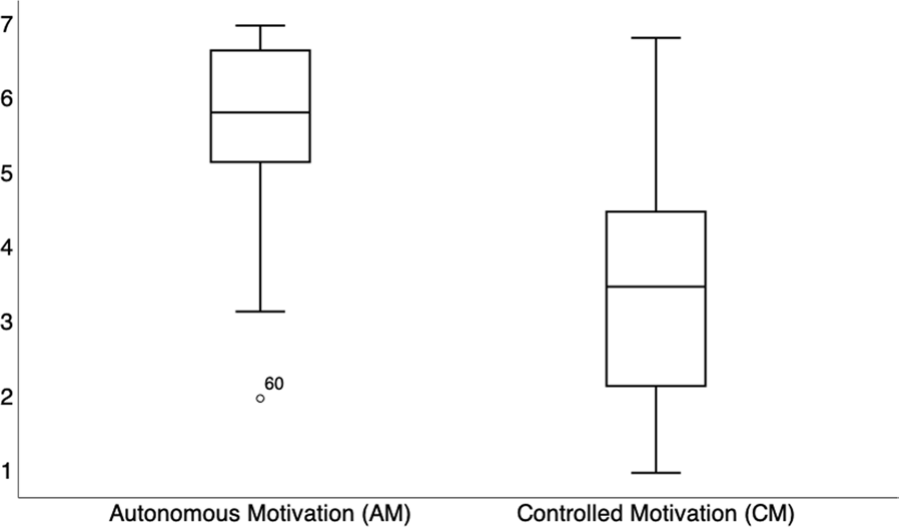
Boxplots depicting the distributions of scores for all participants (*N* = 80) on the AM and CM subscales of the ACMTQ

Scores on EDE-Q and BUT scales indicated clinically significant body discomfort (EDE-Q: total score = 3.43 ± 1.25; BUT: general severity score = 2.44 ± 1.02). Additionally, on the ACMTQ-ITA, AM scores averaged 5.72 ± 1.03, while CM scores averaged 3.36 ± 1.41, as illustrated in Fig. 1’s boxplots.

### Validation of the ACMTQ-ITA

Comprehensive results are summarized in Table 2. Results from the reliability analysis confirmed the internal consistency of the AM factor (α = 0.82, ω = 0.82) and the CM factor (α = 0.76, ω = 0.77). Subsequently, reliability analysis further confirmed the internal consistency of the ACMTQ-ITA overall score (α = 0.79). Reliability analysis further confirmed the internal consistency of the ACMTQ-ITA overall score (α = 0.79). Overall, results from this analysis indicates that the ACMTQ-ITA has high internal consistency.

**Table 2:** Validation of the ACMTQ-ITA

Measures	Autonomous	Controlled
Internal consistency		
Cronbach’s α McDonald’s ω	0.82 0.82	0.76 0.77
Confirmatory factor analysis (CFA)		
Comparative Fit Index (CFI)	0.92	
Akaike Information Criterion (AIC)	3427.26	
Bayesian Information Criterion (BIC)	3486.82	
Root Mean Square Error of Approximation (RMSEA)	0.08 [90% CI 0.04–0.11]	
Standardized Root Mean Square Residual (SRMR)	0.09	

*CI* confidence interval

To follow, the CFA confirmed the two-factor solution (i.e., AM and CM) identified in the original validation of the ACMTQ, as indicated by the set of goodness of fit statistics. In particular, the CFI was 0.90, the AIC was 3484.60, and the BIC was 3544.46. Furthermore, the RMSEA was 0.09 [90% CI 0.055–0.123], and the SRMR was 0.10. Taken together, the values of the above goodness of fit statistics suggest that the proposed two-factor solution adequately accounts for the observed patterns of correlations among the items of the ACMTQ-ITA.

## Discussion

This study aimed to validate an Italian version of the ACMTQ, a readily applicable self-report tool to measure patients’ motivation for treatment in the ED field. In accordance with the original ACMTQ scale, the final version of the ACMTQ-ITA scale is composed of 12 items belonging to two separate subscales (i.e., AM and CM). Further, as for the original validation study, the results of the present research confirmed the psychometric properties of this instrument. Specifically, the scale is reliable, as evidenced by all Cronbach’s α values exceeding 0.70, and it is an adequate measure of motivation for therapy for patients with EDs, as shown by CFA results. Overall, our analyses confirmed the structural two-factor model of the original validation showing a satisfactory internal consistency and acceptable fit.

Patients with EDs often exhibit limited motivation for treatment due to their mixed and ambivalent feelings toward their condition [22]. These individuals often experience conflicting emotions, recognizing the negative consequences of their eating behaviors while also being dependent on the disorder [28]. Eating symptoms can be a dysfunctional but reassuring coping mechanism: the fear of change, uncertainty, and the disorder’s role in their identity can further hinder their motivation to seek treatment [28, 29]. Negative emotions, such as shame and self-hatred, coupled with a lack of insight or denial, can also dampen motivation [22, 30].

In the public health context, careful assessment of motivation for treatment is necessary with a view to allocating resources in the most appropriate way and time [31, 32]. It is indeed appropriate for individuals with lower motivation to receive additional preliminary motivational and psychoeducational sessions before entering regular treatment [33, 34]. Effectively, addressing barriers to treatment motivation, promoting awareness, and fostering support networks are essential instances for improving treatment motivation and ensuring equitable access to care within limited resource environments [35].

Simultaneously, when individuals with EDs are motivated to seek and engage in treatment, it is crucial to initiate early treatment since it significantly increases the likelihood of successful recovery and improved long-term outcomes [36, 37]. Motivated patients are more likely to actively participate in therapy, adhere to treatment recommendations, and make the necessary behavioral changes to promote recovery [38]. They are more open to exploring the underlying emotional and psychological factors contributing to their disorder, and they are willing to challenge their thoughts and beliefs related to food, body image, and self-esteem [38–40]. Treatment motivation also facilitates the development of a strong therapeutic alliance between patients and healthcare professionals, leading to better collaboration and progress.

## Strength and limits

One notable strength of this study lies in its rigorous validation process, which employed reliability analysis with both Cronbach’s α and McDonald’s ω estimates, along with CFA. This comprehensive approach ensures the validity and reliability of the ACMTQ-ITA. Evaluating treatment motivation in EDs is paramount in clinical settings, making the validation of the ACMTQ-ITA particularly significant for informing treatment strategies and interventions.

Some relevant limitations should be acknowledged when interpreting the findings of this study. First, the sample may lack diversity in terms of age, gender, race, ethnicity, socioeconomic background, educational level, and geographic regions, potentially limiting the generalizability of results. Second, the uneven distribution of diagnoses, with higher prevalence rates of AN and BN compared to BED and OSFED/UFED, may not fully represent the diversity of individuals with EDs. Third, the relatively small sample size might affect statistical power and the robustness of results, necessitating caution in generalizing findings. Fourthly, a limitation of the study lies in the choice of translating “treatment” as “trattamento” rather than “terapia” or “percorso di cura,” which may introduce nuances impacting the interpretation and validity of the ACMTQ-ITA in the Italian context. Last, reliance on self-report measures may introduce response bias or social desirability effects, impacting data accuracy. These limitations highlight the importance of future research to address these concerns and enhance the validity and generalizability of findings.

## Conclusion

In conclusion, patients with EDs often struggle with limited motivation for treatment. When individuals with EDs do show motivation to seek and participate in treatment, early intervention becomes crucial, leading to higher chances of successful recovery and improved long-term outcomes. Treatment motivation plays a key role in encouraging active participation, therapeutic alliance, and the exploration of underlying factors contributing to the disorder. The ACMTQ-ITA scale offers a convenient tool to rapidly assess patients’ motivation for treatment in the field of EDs, providing valuable insights that can inform personalized and effective interventions.

## What is already known on this subject?

Treatment resistance is a significant challenge in addressing EDs, and understanding patients’ motivation for treatment is crucial for effective interventions. Previous research has validated the ACMTQ in ED populations, providing a tool to assess treatment motivation. However, the availability of validated measures in different languages, such as Italian, remains limited. This study aimed to address this gap by validating the Italian version of the ACMTQ (ACMTQ-ITA) and evaluating its psychometric properties.

## What this study adds?

This study validates the ACMTQ-ITA as a reliable and valid tool for assessing motivation for treatment in Italian-speaking individuals with EDs. By demonstrating the psychometric properties of the ACMTQ-ITA, this research provides clinicians and researchers with a culturally appropriate instrument to evaluate treatment motivation, enhancing the comprehensiveness and accuracy of assessments in Italian clinical settings.

## Data Availability

The dataset analyzed during the current study are available from the corresponding author on reasonable request.

## Appendix

**Table:**

Autonomous and Controlled Motivation for Treatment Questionnaire (ACMTQ-ITA)											
**Istruzioni** Il presente questionario comprende 12 affermazioni. Si prega di leggere attentamente ciascuna affermazione e assegnare un valore numerico seguendo la scala fornita											
**1** **Fortemente in disaccordo**	**2** **Moderata- mente in disaccordo**	**3** **Lievemente in disaccordo**	**4** **Neutrale**	**5** **Lievemente d’accordo**		**6** **Moderata- mente d’accordo**			**7** **Fortemente d’accordo**		
**Partecipo a questa terapia perché:**				**1**	**2**		**3**	**4**	**5**	**6**	**7**
1. Altre persone si arrabbierebbero con me se non lo facessi				◯	◯		◯	◯	◯	◯	◯
2. Credo personalmente che sia l’aspetto più importante della mia guarigione				◯	◯		◯	◯	◯	◯	◯
3. Gestire il mio disturbo alimentare mi permette di partecipare ad altri importanti aspetti della mia vita				◯	◯		◯	◯	◯	◯	◯
4. Desidero che altri vedano che io riesco a seguire il mio trattamento				◯	◯		◯	◯	◯	◯	◯
5. Ho scelto di rendere il trattamento del mio disturbo alimentare una parte importante della mia vita				◯	◯		◯	◯	◯	◯	◯
6. Mi vergognerei di me stesso/a se non lo facessi				◯	◯		◯	◯	◯	◯	◯
7. Perlopiù voglio che il mio terapeuta pensi che sono un buon paziente				◯	◯		◯	◯	◯	◯	◯
8. Mi sento personalmente soddisfatto/a quando seguo il trattamento del mio disturbo alimentare				◯	◯		◯	◯	◯	◯	◯
9. Mi sentirei in colpa se non facessi ciò che ha detto il mio terapeuta				◯	◯		◯	◯	◯	◯	◯
10. Ho pensato attentamente al trattamento per il mio disturbo alimentare e penso che sia la cosa più importante che io possa fare per stare meglio				◯	◯		◯	◯	◯	◯	◯
11. Essere trattato/a per il disturbo alimentare è una scelta importante che voglio davvero fare per guarire				◯	◯		◯	◯	◯	◯	◯
12. Non voglio che le altre persone siano deluse da me				◯	◯		◯	◯	◯	◯	◯
